# Variation in compensatory strategies as a function of target constriction degree in post-glossectomy speech

**DOI:** 10.1121/10.0009897

**Published:** 2022-04-22

**Authors:** Christina Hagedorn, Yijing Lu, Asterios Toutios, Uttam Sinha, Louis Goldstein, Shrikanth Narayanan

**Affiliations:** 1 Linguistics, College of Staten Island–City University of New York, 2800 Victory Boulevard, Staten Island, New York 10314, USA; 2 Linguistics, University of Southern California, 3601 Watt Way, Los Angeles, California 90089, USA; 3 Viterbi School of Engineering, University of Southern California, 3650 McClintock Avenue, Los Angeles, California 90089, USA; 4 Keck School of Medicine of the University of Southern California, 1975 Zonal Avenue, Los Angeles, California 90033, USA christina.hagedorn@csi.cuny.edu, yijinglu@usc.edu, toutios@gmail.com, sinha@usc.edu, louisgol@usc.edu, shri@ee.usc.edu

## Abstract

Individuals who have undergone treatment for oral cancer oftentimes exhibit compensatory behavior in consonant production. This pilot study investigates whether compensatory mechanisms utilized in the production of speech sounds with a given target constriction location vary systematically depending on target manner of articulation. The data reveal that compensatory strategies used to produce target alveolar segments vary systematically as a function of target manner of articulation in subtle yet meaningful ways. When target constriction degree at a particular constriction location cannot be preserved, individuals may leverage their ability to finely modulate constriction degree at multiple constriction locations along the vocal tract.

## 1. Introduction

Individuals with advanced lingual cancer oftentimes undergo a glossectomy procedure as part of treatment, whereby all or part of the tongue is surgically removed, with or without reconstruction. This surgical treatment may be combined with radiation therapy to improve likelihood of survival. This combined-modality treatment leads to reduced lingual mass and mobility (Pauloski *et al.*, 1998; Nicoletti *et al.*, 2004), giving rise to difficulties in speech production (Imai and Michi, 1992; Bachher *et al.*, 2002). Several studies have investigated speech production in individuals who have undergone glossectomy using a variety of modalities, including acoustics (Logemann *et al.*, 1993; Savariaux *et al.*, 2001; Zhou *et al.*, 2011), electropalatography (EPG) (Fletcher, 1988; Imai and Michi, 1992; Michi, *et al.* 1989), videofluoroscopy (Georgian *et al.*, 1982; Morrish, 1984), ultrasound (Bressmann *et al.*, 2005; Rastadmehr *et al.*, 2008; Acher *et al.*, 2014), cine-MRI (Stone *et al.*, 2004; Stone *et al.*, 2010; Stone *et al.*, 2014a; Stone *et al.*, 2014b; Reichard *et al.*, 2012), and real-time MRI (Hagedorn *et al.*, 2021). Accordingly, individuals who have undergone treatment for lingual cancer have been shown to exhibit impaired lingual dynamics, including reduced lingual range of motion (Bressmann *et al.*, 2005), reduced functional independence of particular segments of the tongue (Stone *et al.*, 2004), and reduced complexity of vocal tract shaping (Hagedorn *et al.*, 2021). Moreover, resection of the oral tongue, as compared to the base of tongue, has been observed to most negatively impact post-operative speech intelligibility, and speech sounds requiring coronal constrictions are most likely to be impaired (Imai and Michi, 1992; Wong *et al.*, 2007; Bhattacharya *et al.*, 2021; Bressmann *et al.*, 2009).

Numerous studies over the past several decades have demonstrated that in response to this structural perturbation to the motor speech system, individuals frequently utilize compensatory strategies to produce consonants post-operatively, recruiting articulators other than those typically used. For example, when the tongue tip has been resected, a variety of compensatory strategies may be used for the production of target coronal stops /t/ and /d/, including retracting and pressing the lower lip against the alveolar ridge (Amerman and Laminack, 1974; Duguay and Flach, 1964; Brodnitz, 1960; Massengill *et al.*, 1970; Skelly *et al.*, 1971), elevating the lower lip behind the upper teeth (Duguay and Flach, 1964), and making palatal contact with the entire tongue body (Öberg, 2009). Similarly, compensatory strategies observed in the production of target coronal fricatives include bilabial approximation (Skelly *et al.*, 1971; Fletcher, 1988), recruitment of the tongue body to assist the tip in forming the fricative constriction (Reichard *et al.*, 2012), and substantial retraction and depression of the tongue tip (a pattern not frequently exhibited by typical speakers) (Stone *et al.*, 2014a; Stone *et al.*, 2014b).

Compensatory behavior observed is not limited to replacement of a target constriction (e.g., of the tongue tip) with one formed by an alternate articulator, or set of articulators (e.g., the lips). Rather, it may involve simultaneous production of two constrictions, one of which may be a full or partial production of the target gesture. For example, in place of target coronal stops /t/ and /d/, individuals may produce a “flicker-like” motion of the lips along with partial tongue tip elevation (Aanya *et al.*, 2020), or a lingual gesture with varying degrees of labial protrusion and retraction (Georgian *et al.*, 1982). The existing literature also suggests that individuals may also modulate subtle aspects of relative timing of the compensatory gestures. Georgian *et al.* (1982) describes compensation for some target alveolar oral stops as initial velar contact followed quickly by palatal contact, while Skelly *et al.* (1971) describes compensation for some target alveolar nasal stops as momentary velar contraction followed by quick uvular relaxation with the lips approaching closure.

The aforementioned findings provide critical clinical and theoretical insight, illustrating the ability of the motor speech system to adapt to anatomical and physiological perturbation to the tongue. However, no research thus far has focused on how compensatory strategies used by patients may vary depending on target manner of articulation for target segments that require constriction formed primarily by a single articulator (e.g., the tongue tip) at a single place of articulation (e.g., alveolar). That is, when the tongue tip is no longer present or able to function as it was pre-operatively, are segments that vary in manner of articulation, yet require a substantial coronal gesture, compensated for using similar strategies? Or, do the compensatory patterns observed vary as a function of target manner of articulation?

In this pilot study, we use real-time magnetic resonance imaging (rtMRI) and a semi-automatic method of identifying both constriction location and constriction degree in vocal tract images during target constrictions. As described above, a variety of methods and imaging modalities, ranging from clinical observation to quantitative articulometry and palatography, have been utilized to investigate compensatory behavior in post-glossectomy speech. rtMRI, however, is particularly well-suited for this purpose, given that it provides a full midsagittal view of the vocal tract from the lips to the larynx, without exposing the participant to ionizing radiation. In doing so, it is able to fully capture constrictions formed by multiple articulators, at any point along the vocal tract, at once, in contrast to other methods, such as ultrasound or electropalatography, which quantitatively capture only lingual constrictions. An additional merit of rtMRI as compared to other articulometry modalities (e.g., electropalatography, electromagnetic articulography, etc.) is that it does not require intraoral devices or adhesion of sensors to articulators that may be tender or swollen post-surgically.

The aim of this pilot study is to determine whether compensatory strategies used by individual speakers who have undergone partial glossectomy vary systematically as a function of target manner of articulation. We predict that within individual speakers, compensatory strategies for target alveolar segments that vary in manner of articulation will differ, given that though they share a single primary target articulator (i.e., the tongue tip), each is associated with distinct target articulatory configurations and aerodynamic, acoustic, and perceptual characteristics that may influence which compensatory strategy is deemed most effective by the motor speech system for production of that particular target segment. Specifically, we predict that target alveolar oral stops (i.e., /t/, /d/), which require a complete circumpalatal seal, are most likely to be compensated for by individuals with reduced anterior lingual mass and mobility. We predict that target alveolar fricatives (i.e., /s/, /z/) may be deemed achievable by the post-operative motor speech system, and therefore may not be produced compensatorily, or with as complex compensatory patterns. Although individuals may exhibit difficulty creating the simultaneous anterior coronal constriction and tight side contact seal with the teeth necessary to produce high-intensity, high-frequency sibilant noise, resulting in leakage and therefore weakened sibilance, creating turbulence of some kind may still be feasible. Likewise, target alveolar nasal stops may not be regularly compensated for, given that even in the absence of a complete circumpalatal seal (resulting in a slight degree of oral airflow) a nasal percept can be produced, particularly if velopharyngeal port function is intact. Compensation may not be deemed necessary for production of lateral approximant /l/, given that although complete coronal constriction is typically required, no circumpalatal seal is targeted; rather, the lateral margin(s) of the tongue are lowered. Producing this articulatory configuration may be possible for post-operative speakers, despite reduced lingual mass and mobility.

## 2. Method

### 2.1 Participants

The participants in this pilot study were two monolingual speakers of American English, 1 male [M1], age 70, and 1 female [F1], age 52, who had undergone partial glossectomy with reconstruction and post-operative chemo-radiation therapy. M1 underwent resection of a >6 cm (T4) tumor in the oral tongue region, while F1 underwent resection of a 4–6 cm (T3) tumor in the oral and base of tongue regions, and neither had dental involvement. Both participants underwent lingual reconstruction using a radial forearm free flap. Each of the participants acknowledged speech production difficulties. However, due to extenuating circumstances, neither received speech or swallowing therapy between the time of treatment and the time of the data collection scan, which took place at least 6 months post-operatively.

The speech of each participant was scored by three licensed speech and language pathologists, independently, using the American Speech-Language-Hearing Association (ASHA) National Outcomes Measurement System (NOMS) Motor Speech sub-assessment to derive general indices of participants' functional communication as it relates to the motor speech system. The level of speech impairment was comparable for each participant, and a high level of interrater reliability was exhibited (M1: M = 4.3; SD = 0.57; F1: M = 4; SD = 0). A NOMS Motor Speech score of 4 reflects that “in simple structured conversation with familiar communication partners, the individual can produce simple words and phrases intelligibly. The individual usually requires moderate cueing in order to produce simple sentences intelligibly, although accuracy may vary.” Acoustic speech samples for each participant are provided as supplemental material.^1^

### 2.2 Stimuli

Stimuli included sentences from the TIMIT corpus (Wrench and William, 2000) and excerpts from the Rainbow Passage (Appendix), displayed to the participants using a projection and mirror setup.

### 2.3 Procedure

Image data were acquired on a 1.5 T GE Sigma scanner, using a 13-interleaf spiral gradient echo pulse sequence (TR = 6.004 ms, FOV = 200 × 200 mm, flip angle = 15º) and a custom 4-channel head and neck receiver coil. Pixel density in the midsagittal plane (5 mm slice thickness) was 84 × 84 (2.38 × 2.38 mm^2^). Image data were acquired at a rate of 12.8 frames per second and reconstructed at 23.79 frames per second using a sliding window technique. Acoustic data were recorded inside the scanner at 20 kHz simultaneously with MRI image acquisition, and noise reduced using the custom protocol described in Bresch *et al.* (2006).

### 2.4 Articulator segmentation of real-time MRI data and semi-automatic identification of constriction location and constriction degree

**Fig. 1.**
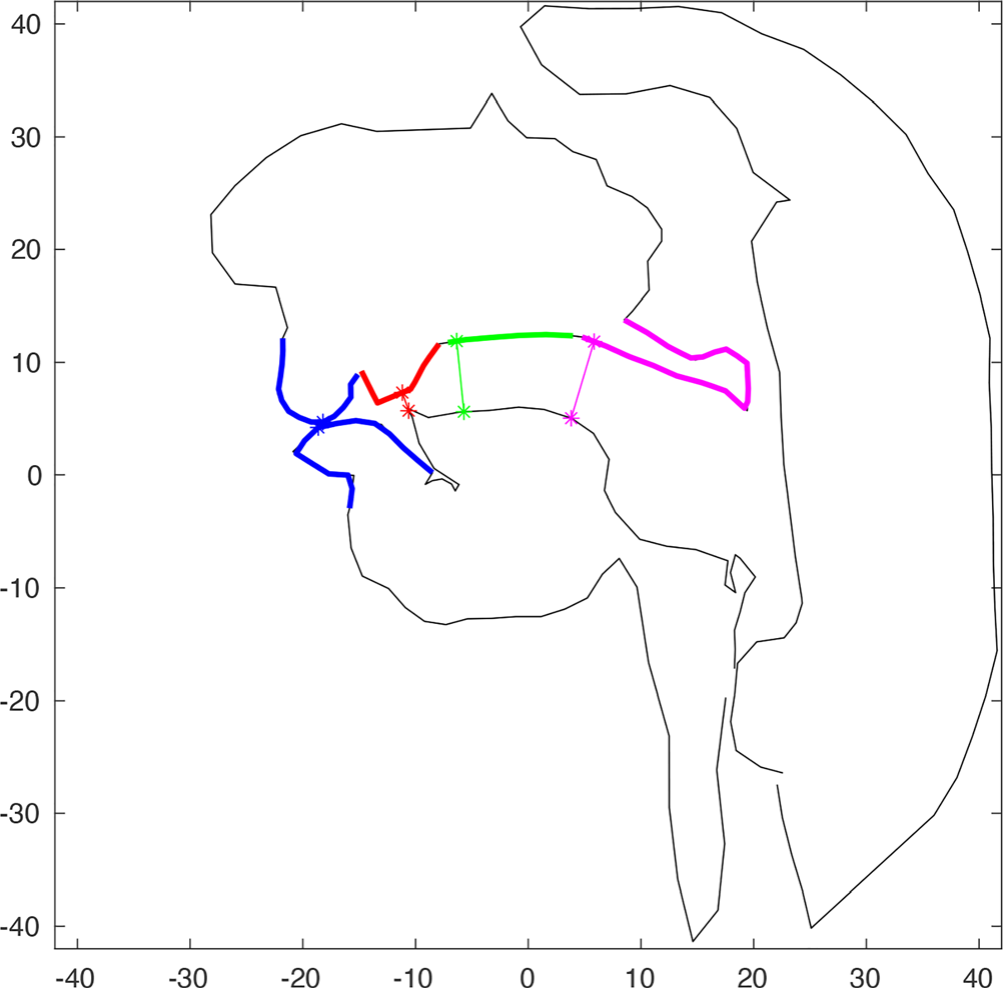
Labial (blue), alveolar (red), palatal (green), and velar (pink) regions within which constriction locations and degrees are identified.

First, fine-grained, manual, frame-by-frame analysis was completed for all targeted speech segments. Working alongside the time-aligned acoustic signal, frame number corresponding to maximum constriction, regardless of constriction location, was logged for all targeted consonants. Then, all image data were segmented along air-tissue boundaries, based on location and connection of intensity thresholds, as described in Bresch *et al.* (2008). Constriction locations were defined using a two-step process. First, labial, alveolar, palatal, and velar regions were identified for each speaker, and within each region, all possible cross-distances (in pixels) were calculated, and coordinates along the upper margin of the vocal tract (i.e., the upper lip, alveolar ridge, hard and soft palates) at which minimum cross-distances occur were determined, resulting in a location of maximum constriction (minimum cross-distance) within each region (Fig. 1). Constriction location(s) for a given segment were defined as those regions containing coordinates at which constriction degree fell below 1.5 pixels (3.57 mm). Constrictions corresponding to target segments of interest, on all frames previously identified as corresponding to maximum constriction, were then classified as involving full occlusion (<0.5 pixel), partial occlusion (0.5–1 pixel), or marginal occlusion (1.001–1.5 pixels). Frames of maximum constriction were confirmed as such by assessing and comparing constriction degrees associated with immediately adjacent frames. Productions corresponding to target consonant clusters were excluded from analysis, given the high degree of co-articulation expected to be exhibited in the production of the required constrictions. Given that this study aims to investigate compensation for target coronal constrictions, and given well-documented asymmetries between the production of /l/ in onset and coda positions concerning coronal constriction, only productions of target /l/ in onset position were included in the analysis. Additionally, since multiple previous studies have noted the use of the lips in compensatory behavior (Skelly *et al.*, 1971; Georgian *et al.*, 1982; Fletcher, 1988; Aanya *et al.*, 2020), target labial oral and nasal stop segments were included in analysis for comparison to any possible labial compensatory behavior observed for target alveolar segments.

## 3. Results

### 3.1 Speaker F1

*Target alveolar segments*. In the production of target voiceless alveolar stop /t/, F1 most frequently produces full or partial occlusion at the labial constriction location, with some (variable) degree of occlusion at the alveolar constriction location (75%, 15 of 20 tokens). Full occlusion is produced in the labial region with *no* alveolar constriction in low vowel contexts, comprising 20% of tokens (4 of 20 tokens). In the production of target voiced alveolar stop /d/, F1 most frequently produces full occlusion in the labial region (85.7%, 6 of 7 tokens), at times with some degree of occlusion in the alveolar region (42.9%, 3 of 7 tokens) (supplemental figure 1A).^1^ As in the case of /t/, full occlusion of the lips without alveolar constriction occurs in low vowel contexts, comprising 57.1% of tokens (4 of 7 tokens).

**Table 1.** Speaker F1 co-produces labial and alveolar constrictions to compensate for alveolar oral stops, fricatives, nasal stops, and lateral approximants.

COMPENSATORY PATTERNS OF SPEAKER F1 (ORAL AND BASE OF TONGUE RESECTION)				
	ALVEOLAR SEGMENTS COMPENSATED FOR			
	ORAL STOPS	FRICATIVES	NASAL STOPS	LATERAL APPROX.
LABIAL	Full/Partial	Full/Partial	Variable	Full/Partial
ALVEOLAR	Variable	Variable	Variable	Variable

In the production of target voiceless alveolar fricative /s/, F1 produces full or partial occlusion in the alveolar region, with partial or marginal occlusion in the labial region (71.4%, 5 of 7 tokens) (supplemental figure 1B).^1^ Occasionally, occlusion is formed in only one of these regions (28.6%, 2 of 7 tokens). In the production of target voiced alveolar fricative /z/, F1 produces full, partial, or marginal occlusion in the alveolar region (100%, 7 of 7 tokens), with some degree of occlusion in the labial region (71.4%, 5 of 7 tokens). Occasionally, occlusion is formed in only the alveolar region (28.6%, 2 of 7 tokens).

In the production of target alveolar nasal stops, F1 most frequently produces full, partial, or marginal occlusion in the labial region (94.7%, 18 of 19 tokens), with some degree of occlusion in the alveolar region (78.9%, 15 of 19 tokens) (supplemental figure 1C).^1^ Full occlusion in only the labial or alveolar region occurs infrequently (21.1%, 4 of 19 tokens).

In the production of target lateral approximants, F1 most frequently produces full or partial occlusion in the alveolar region (73.3%, 11 of 15 tokens), at times with some degree of occlusion in the labial region (26.6%, 4 of 15 tokens) (supplemental figure 1D),^1^ primarily in the context of rounded vowels.

*Target bilabial segments*. In the production of target voiceless bilabial oral stop /p/, F1 most frequently produces full occlusion in the labial region (supplemental figure 1E^1^) (71.4%, 5 of 7 tokens), and sometimes produces partial occlusion in the labial region (28.6%, 2 of 7 tokens). To produce target voiced bilabial oral stop /b/, F1 produces full occlusion (66.6%, 6 of 9 tokens) and partial occlusion (33.3%, 3 of 9 tokens) in the labial region.

In the production of target bilabial nasal stops, F1 invariably produces full occlusion in the labial region (supplemental figure 1F^1^) (100%, 5 of 5 tokens).

In sum, F1 co-produces labial and alveolar constrictions of varying magnitude to compensate for target alveolar segments (Table 1).

**Fig. 2.**
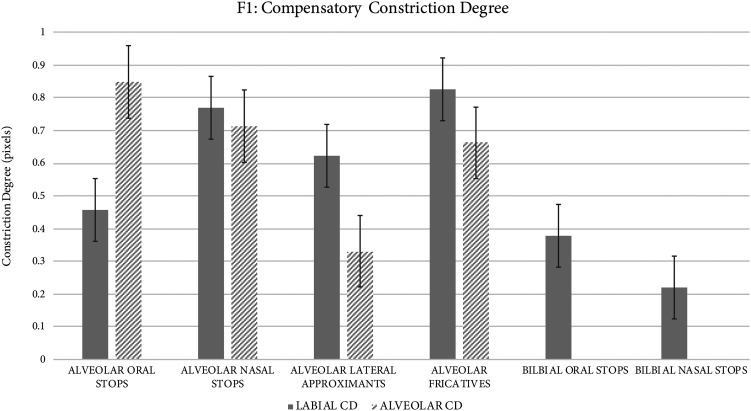
Compensatory constriction degree patterns vary systematically for distinct target manners of articulation. Target bilabial oral stops are produced with labial constriction degrees comparable to those produced compensatorily for alveolar oral stops.

*Compensatory constriction degree.* In addition to considering where compensatory constrictions of varying degrees are formed for target segments differing in manner of articulation, finer-grained comparison of constriction degree (where constrictions were identified) with which compensatory constrictions are produced reveals differences across target segment types. As reflected in Fig. 2, F1 produces target alveolar stops with relatively narrow labial constrictions (0.46 pixels) and relatively wide alveolar constrictions (0.85 pixels). Nasal stops are compensated by creating similar constriction apertures in both labial and alveolar regions that are comparable, in degree, to those used in fricative production (0.77 pixels and 0.71 pixels, respectively). Lateral approximants are compensated for using a constriction aperture pattern opposite of that exhibited for oral stops; a very narrow constriction is formed in the alveolar region (0.33 pixels), while a wider constriction is formed in the labial region (0.62 pixels). Alveolar fricatives are compensated for using a somewhat wider constriction in the labial region (0.83 pixels) than in the alveolar region (0.66 pixels). The labial constrictions used for target labial oral and nasal stops are somewhat narrower than those used compensatorily in the production of target alveolar oral stops, and substantially more narrow than those used in the production of target alveolar nasal stops, laterals, and fricatives.

### 3.2 Speaker M1

*Target alveolar segments*. In the production of target voiceless alveolar stop /t/, M1 invariably produces full occlusion in the velar region, with some degree of occlusion in the labial and palatal regions (100%, 8 of 8 tokens). In the production of target voiced alveolar stop /d/, M1 produces full occlusion in the velar region with some degree of occlusion in the labial and palatal regions (100%, 2 of 2 tokens) (supplemental figure 2A).^1^

**Table. 2.** Speaker M1 co-produces labial and velar constrictions during production of target alveolar oral stops, fricatives, nasal stops, and lateral approximants.

COMPENSATORY PATTERNS OF SPEAKER M1 (ORAL TONGUE RESECTION)				
	ALVEOLAR SEGMENTS COMPENSATED FOR			
	ORAL STOPS	FRICATIVES	NASAL STOPS	LATERAL APPROX.
LABIAL	Variable	Marginal	Variable	Partial/Marginal
VELAR	Full	Full	Full	Full

In the production of target voiceless alveolar fricative /s/, M1 produces full occlusion in the velar region, with marginal occlusion in the labial region (100%, 3 of 3 tokens) (supplemental figure 2B).^1^ In the production of target voiced alveolar fricative /z/, M1 produces full occlusion in the velar region, with marginal occlusion in the labial region (100%, 2 of 2 tokens).

In the production of target alveolar nasal stops, M1 produces full occlusion in the velar region (100%, 6 of 6 tokens), with partial or marginal occlusion in the labial region (66.6%, 4 of 6 tokens) (supplemental figure 2C).^1^ Occasionally (33.3%, 2 of 6 tokens), M1 produces full labial and velar occlusion.

In the production of target lateral approximants, M1 produces full occlusion in the velar region, with partial or marginal occlusion in the labial region (supplemental figure 2D^1^) (100%, 7 of 7 tokens).

*Target bilabial segments*. In the production of target voiceless bilabial oral stops, M1 produces full labial occlusion and full velar occlusion (100%, 1 of 1 token) (supplemental figure 2E).^1^ In the production of target voiced bilabial oral stops, M1 produces full velar occlusion (100%, 3 of 3 tokens) with partial labial occlusion (66.6%, 2 of 3 tokens) or full labial occlusion (33.3%, 1 of 3 tokens).

In the production of target bilabial nasal stops, M1 produces full or partial velar constriction with full or partial labial constriction (supplemental figure 2F^1^) (100%, 3 of 3 tokens).

In sum, M1 invariably produces a full velar constriction, regardless of target segment type, along with a labial constriction of varying magnitude, for target alveolar segments (Table 2).

**Fig. 3.**
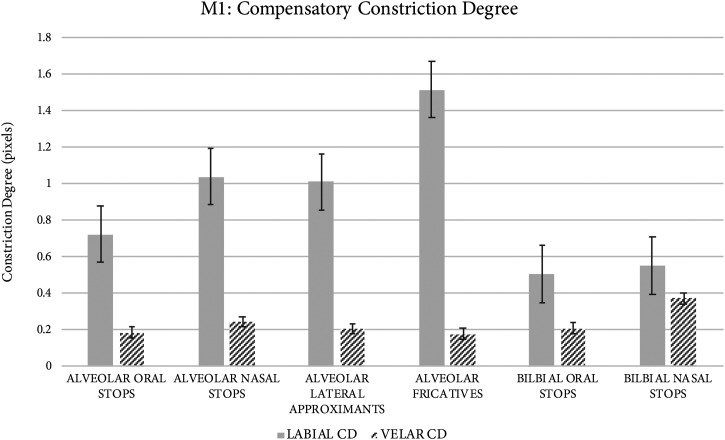
Constriction degree formed by the lips, in particular, varies for segments differing in target manner of articulation. Target bilabial stops are produced using constrictions in the velar and labial regions; target labial constriction degree is narrower than for compensatory labial constrictions produced for target alveolar oral stops.

*Compensatory constriction degree.* As illustrated in Fig. 3, constriction degree in the velar region does not vary substantially across segments differing in target manner of articulation. However, constriction degree in the labial region does vary by target manner of articulation. Target alveolar oral stops are produced compensatorily with the narrowest labial constriction (0.72 pixels), while target alveolar nasal stops and laterals are produced with wider labial constriction (1.04 pixels and 1.01 pixels, respectively). Target alveolar fricatives are produced with the widest labial constriction (1.5 pixels). Both labial and velar constrictions are present during target labial oral and nasal stops. The labial constriction degree produced during target labial stops is slightly more narrow than the labial constriction degree produced compensatorily during target alveolar stops, and substantially more narrow than those produced during target alveolar nasals tops, laterals, and fricatives.

## 4. Discussion

Overall, both F1 and M2 exhibit compensatory behavior for the production of target alveolar constrictions. This is expected, given that each has undergone glossectomy affecting the oral tongue.

The data reveal that in the production of nearly all target alveolar segments, regardless of target manner of articulation, F1 frequently utilizes simultaneously produced alveolar and bilabial constrictions. However, patterns in the degree to which these constrictions are produced vary systematically across target segment type. In the production of target alveolar oral stops, F1 typically produces full or partial labial occlusion with variable degrees of alveolar occlusion. The degree of compensatory labial occlusion for oral alveolar stops is comparable to that produced in target labial oral stops. In contexts of low back vowel /ɑ/ only, F1 produces no alveolar constriction, likely due to difficulty reaching the alveolar region with the residual tongue tip caused by the low and back starting position of the tongue body. On average, the labial constrictions produced are substantially more narrow than the alveolar constrictions produced. This particular articulatory configuration likely results in an approximation of the acoustic and perceptual correlates of target alveolar oral stops; the narrow labial constriction gives rise to the period of acoustic silence, while the simultaneously produced partial alveolar constriction, oftentimes wide in constriction degree, contributes to the production of alveolar-like formant transitions into and out of adjacent vowels. F1's co-production of a labial constriction and a slightly narrower alveolar constriction for target alveolar fricatives likely gives rise to an acoustic percept associated with frication (i.e., turbulent airflow), despite strong sibilance not likely being produced. F1 compensates for the target alveolar constriction in nasal /n/ by co-producing labial and alveolar constrictions of comparable constriction degrees. Notably, despite the target forms of each requiring full oral occlusion in the alveolar region, the constrictions formed are substantially wider than the primary labial constriction used to compensate for target alveolar oral stops. It is plausible that this configuration, despite possibly not involving complete oral occlusion (i.e., via a complete circumpalatal seal) at all, results in a perceptually acceptable target alveolar nasal segment. In F1's production of the target alveolar lateral, substantial constriction in the alveolar region is formed using the tongue tip, with a constriction degree more narrow than any other used in target alveolar segments. Wide and variable labial constriction is observed in contexts of rounded vowels only, and may arise due to co-articulation and not be part of the compensatory process for this speaker. That F1 produces target laterals using the tongue alone, in contrast to other target alveolar segments for which she compensates using co-production of labial and alveolar constrictions, is notable. It is plausible that despite her reduced lingual mass and mobility, forming a complete coronal constriction at the alveolar ridge in the midline of the vocal tract (in the midsagittal MRI view) is possible precisely *because* complete occlusion is not made at the lateral palatal margins during target /l/; more lingual tissue can be dedicated to forming a complete constriction at a single alveolar point at the vocal tract midline *because* no lingual tissue is recruited to form complete occlusion in lateral portions of the alveolar or palatal regions.

Interestingly, a small portion (5 of 16 tokens) of F1's target labial oral stop productions involve partial occlusion rather than full. The possibility that this reflects slight labial impairment caused by radiation-induced fibrosis cannot be definitively ruled out without comparison to pre-operative data. However, given that all such tokens occur in co-articulatory contexts that would facilitate wider stop constriction apertures being formed (e.g., intervocalically, preceding low vowels, preceding a rounded segment), and given that F1 invariably forms full labial occlusion in the production of target bilabial nasal stops, it is far more likely that this pattern does not reflect labial impairment.

During the production of target alveolar segments, M1 exhibits co-produced labial constrictions and velar constrictions. For all target alveolar segments, full velar constrictions are formed along with labial constrictions of varying degrees, depending on segment type. Strikingly, velar constrictions are also produced by M1 during target bilabial oral and nasal stops. This suggests that rather than the velar constriction being used strategically as a compensatory mechanism, it more likely emerges as a physiological result of the additional lingual mass caused by the reconstructive lingual flap in the anterior oral tongue region (supplemental figure 2),^1^ particularly when the jaw position is high due to consonantal constrictions. While perturbation following glossectomy and reconstruction is frequently observed to result in articulatory difficulty due to *insufficient* constriction formation, these data demonstrate that, conversely, it may also cause *forced* constriction formation due to increased lingual mass, impaired lingual mobility, or some combination thereof. This forced constriction, though not initially formed to compensate for challenging segments, may be taken into account by the post-operative motor speech system when selecting possible compensatory constrictions to be formed alongside the forced constriction (e.g., those involving the lips).

Target oral stops /t/ and /d/ are produced by M1 with the narrowest labial constriction. The co-production of velar and labial occlusions contributes to the period of acoustic silence produced. Target alveolar fricatives /s/ and /z/ are produced by M1 using relatively wide labial constriction in conjunction with the narrow velar constriction observed in the midsagittal plane. It is possible that velar occlusion is made only in the midsagittal plane, but that airflow is permitted laterally, to effect frication even in the absence of sibilance. Target nasal stop /n/ is produced with a slightly wider labial constriction than is produced for target alveolar oral stops, along with narrow velar constriction. Like for target alveolar fricatives, it is plausible that full occlusion is made in the midsagittal plane, though not laterally (precluding a full circumpalatal seal). Despite this, perceptually acceptable target alveolar nasal resonance may be produced.

Target lateral /l/ is produced by M1 using the widest labial constriction of all target alveolar segments, along with the narrow velar constriction. The labial constriction formed may compensate for the lack of tongue dorsum retraction typically produced during target /l/; both of these mechanisms would effectively shorten the posterior portion of the oral cavity relative to the anterior portion, and consequently lower all resonant frequencies.

As in the case of F1, M1's production of target labial stops sometimes involves partial constriction, particularly in co-articulatory contexts that facilitate the production of wider constriction apertures.

All in all, the pilot data at hand illustrate that the compensatory strategies used by individuals who have undergone oral tongue glossectomy to produce target alveolar segments do vary systematically as a function of target manner of articulation in subtle yet meaningful ways. When target constriction degree at a particular constriction location cannot be preserved, patients may leverage their ability to finely modulate constriction degree at multiple constriction locations along the vocal tract to compensate. Additionally, individuals who have undergone glossectomy with reconstruction may exhibit articulatory challenges caused by forced vocal tract constriction formation, as in the case of M1, in addition to the challenges related to insufficient constriction formation that are more traditionally acknowledged and observed. Moreover, this pilot study demonstrates that simultaneously produced constrictions which are unlikely to be captured by impressionistic clinical observation or other imaging modalities are able to captured using real-time MRI. The utility of the particular analytical technique employed in this pilot study has also been demonstrated; whereas methods relying on observing articulatory behavior of independently chosen articulatory flesh-points or at constriction locations defined *a priori*, the method at hand ensures that constriction location and degree are identified in a data-driven way. This is particularly important when analyzing data of individuals who are likely to use articulatory patterns other than those used by typical speakers.

While this pilot study has helped further characterize possible compensatory behaviors in post-glossectomy speech, it is not without limitations. Most notably, this study relied on data from only two speakers, precluding one from making generalizations about compensatory behaviors of this population based on the patterns observed. Moreover, the speech sample produced by speaker M1 was particularly limited in duration, rendering relatively few analyzable tokens of interest. Future studies would, ideally, include larger participant populations, especially given the substantial amount of interspeaker variability that is expected to be exhibited in patient populations, as well as more extensive speech samples, despite scanner time oftentimes being limited due to patient discomfort or fatigue. Additionally, while our study did not include pre-operative data collection or analysis due to the prioritization of surgery immediately following diagnosis, collecting pre-operative data as well as post-operative data at multiple time points longitudinally would allow for statistical analysis of articulatory changes over time. However, it is important to note that pre-operative speech samples are not likely to be representative of individuals' typical speech, due to the structural perturbation caused by the tumor and discomfort associated with it (Zhou *et al.*, 2011). Future studies will also benefit from recent developments in data acquisition technology allowing for data to be collected in multiple planes (e.g., coronal and axial, in addition to sagittal) and in 3 dimensions (Kim *et al.*, 2009; Lim *et al.*, 2019), and for complexity of vocal tract shaping due to lingual movement in these planes to be quantified (Hagedorn *et al.*, 2021). Using these methods will enable researchers to determine whether complete occlusion in the midsagittal plane (as observed in the present data) is accompanied by complete or partial occlusion in lateral regions, as well as to examine patterns in groove shaping during the production of other segments, including target alveolar fricatives. Recent developments in real-time MRI scanner technology and denoising tools (Vaz *et al.*, 2018) will allow for higher quality acoustic data to be collected, facilitating acoustic analysis. Access to reliable acoustic data will enable researchers to determine whether theory-based models used to simulate post-glossectomy speech produce acoustic output that aligns with the patient data.

The constriction identification and categorization approach that we use is advantageous in that it does not rely on *a priori* assumptions regarding constriction location and degree. However, fine-grained interpretation of findings based on this approach would benefit from its application to large amounts of data from typical speakers, in the future. This will allow researchers to classify constriction degrees for a given target segment as “typical” or “atypical” in a data-driven way, based on comparison to patterns exhibited by the typical population.

Last, the findings of this pilot study have clinical implications and can be used to inform and refine intervention strategies for individuals who have undergone treatment for lingual cancer. First, they showcase compensatory strategies involving “double articulations” used by patients post-operatively that would not likely be observable in a clinical setting due to posterior constrictions being occluded by anterior constrictions. While the patients in our study exhibited these strategies spontaneously (i.e., not under the direction of a clinician) to produce fairly intelligible speech, patients who do not develop compensatory strategies spontaneously may benefit from being introduced to this approach as part of their speech rehabilitation plan, alongside the more commonly recommended strategies of “over articulation” and speaking rate reduction. Second, the findings of our study demonstrate that compensatory strategies used by the participants vary systematically based on target manner of articulation; target segments that do not require formation of a circumpalatal seal or build-up of intraoral air pressure are not likely to be compensated for in the same way as those that do. Clinicians may incorporate this into their practice by suggesting distinct articulatory patterns for target segments differing only in target manner of articulation, rather than making across-the-board strategy recommendations based on target place of articulation or primary articulator used. Such an approach would benefit patients by enhancing the perceptual contrast produced for segments sharing a target place of articulation.

## Footnotes

^1^
See supplementary material at https://www.scitation.org/doi/suppl/10.1121/10.0009897 for rtMRI frames depicting compensatory behaviors for each speaker and an acoustic speech sample from each speaker.

## Appendix

Phrases read by participants.

TIMIT Sentences:

She had your dark suit in greasy wash water all year.

Don't ask me to carry an oily rag like that.

Rainbow Passage Excerpt:

When the sunlight strikes raindrops in the air, they act like a prism and form a rainbow. The rainbow is a division of white light into many beautiful colors. These take the shape of a long round arc with its path high above and its two ends apparently beyond the horizon.
